# Patient-Initiated Follow-Up (PIFU) as reorganized support for increased patient involvement – focus group discussions among patients’ with inflammatory arthritis

**DOI:** 10.1186/s41927-020-00143-6

**Published:** 2020-06-30

**Authors:** Bianca Bech, Jens Jørgen Lykkegaard, Tine Lundbak, Heidi Morsø Schrøder, Line Mette Birkeland, Mette Lund Schlyter, Lotte Hanne Hansen, Lillian Dalsgaard, Bente Appel Esbensen

**Affiliations:** 1 Center for Rheumatology and Spine Diseases, Centre for Head and Orthopaedics, Rigshospitalet, Valdemar Hansens Vej 17, Indgang 5, stuen, 2600 Glostrup, Denmark; 2 Copenhagen Center for Arthritis Research (COPECARE), Center for Rheumatology and Spine Diseases, Centre of Head and Orthopaedics, Rigshospitalet, Glostrup, Denmark; 3 Patient Research Partner, Copenhagen, Denmark; 4 Department of Clinical Medicine, Faculty of Health and Medical Sciences, University of Copenhagen, Copenhagen, Denmark

**Keywords:** Dialogue, Disease management, Nurse-led outpatient clinic, Nurse’s role, Open-access, Patient experiences, PIFU, Practice patterns, Responsibility, Rheumatology

## Abstract

**Background:**

Inflammatory Arthritis is characterized by lifelong medical treatment and an unpredictable trajectory because of the fluctuating nature of the diseases. Proactive disease management is recommended, which includes close monitoring of disease activity that traditionally has been ensured by outpatient visits to rheumatologists at various fixed intervals. Internationally, there is a growing interest in how healthcare systems can be more flexible, individual-oriented and increasingly involve patients with lifelong diseases in their own treatment and care. We aimed to explore how patients with Inflammatory Arthritis with low disease activity or remission (DAS-CRP < 2.9) experience patient involvement in a reorganized follow-up care based on flexibility and patient-initiated contact.

**Methods:**

We conducted a qualitative study based on four mixed group discussions focused on patients with inflammatory arthritis (rheumatoid arthritis [*n* = 21], axial spondyloarthritis [*n* = 3] and psoriatic arthritis [*n* = 1]) participating in a reorganized follow-up care. Changes in follow-up included access to a nurse and patient-initiated follow-up (PIFU). The analysis was based on content analysis. The reporting adheres to the Consolidated Criteria for Reporting Qualitative Research (COREQ).

**Results:**

In total, 25 patients (20 females (80%), mean age 61.8 [range 28–79]) participated. We identified three categories. 1) **Patient-Initiated Follow-Up do not affect patients’ perceived support in disease control**; this refers to patients’ experience of more time available through better resource utilization, as well as trust that access to professional support would be available whenever needed. The category 2) **Information is valued by patients to delineate responsibilities in a new patient role** reflects patients’ uncertainty in the transition to PIFU, combined with confusion about the distribution of responsibilities. 3) **Patients need both extended perspectives of their arthritis and focused dialogue** is about expanding patients’ understanding of their arthritis by interaction over time with both a rheumatologist and a rheumatology nurse in a focused dialogue to involve the patient.

**Conclusions:**

Patients participating in PIFU welcome the flexibility and involvement. However, patients need relevant information to act adequately within a new patient role. Interaction with both rheumatologists and nurses, combined with sufficient time for dialogue, broaden patients’ perspective, make opportunities for action visible, and contribute to patients’ ability to participate in follow-up care.

## Key Messages

- What is already known about this subject?

In Denmark, patients with inflammatory arthritis ask for flexible solutions and opportunities to be involved in their treatment and care. Internationally, various initiatives have been implemented during the past decade, with increased nurse involvement, which is supported by research. It shows that nurse-led follow-up is as safe as conventional rheumatologist-led follow-up in patients with low disease activity or remission.

- What does this study add?

Patients with Inflammatory Arthritis with low disease activity or remission (DAS-CRP < 2.9) acknowledge reorganized follow-up care; however, adequate information is required to take on new responsibilities in the patient role. Patients benefit from consultations with both a rheumatologist and a rheumatology nurse.

- How might this impact on clinical practice?

The future arrangements of outpatient clinics should ensure that patients have the opportunity to consult with both a rheumatologist and a nurse, which would lead to patient involvement in a broader perspective in relation to arthritis, for both patients and health professionals.

## Background

Inflammatory arthritis (IA) covers rheumatoid arthritis (RA), axial spondyloarthritis (axSpA) and psoriatic arthritis (PsA). IA is characterized by lifelong medical treatment and an unpredictable trajectory because of the fluctuating nature of the diseases [1]. Internationally, a treat-to-target strategy for proactive management of IA is recommended, which includes continuous monitoring of the diseases supported by longitudinal patient-reported outcomes [1–4]. Close monitoring of disease activity has traditionally been ensured by outpatient visits to rheumatologists at various fixed intervals [1, 5, 6]. A large proportion of the fixed controls do not result in changes [7]. At the same time, timely access and necessary interventions are a challenge due to patients’ fluctuating needs.

Almost 50 years ago it was shown in a randomized controlled trial (RCT) that other health professionals in rheumatology (HPRs) such as occupational therapists could also undertake close disease monitoring among patients with RA [8]. Further evidence on the doctor-nurse overlap highlighted a yet unrealized scope in many systems to extend the utilization of nursing skills [9, 10]. Recently, a meta-analysis confirmed that nurse-led follow-up resulted in equivalent or improved control of disease activity in outpatients with RA, compared to physician-led follow-up, and without differences in clinical and psychological factors [11]. In addition, nursing consultations can improve patient self-efficacy, and increase self-esteem and patient satisfaction [12–14]. Furthermore, qualitative studies of patients with RA have indicated that nursing involvement contributes to both empowerment of the patient and increased patient autonomy, both of which are expected to have a positive effect on lifelong disease management [15, 16].

Also, leaving the initiation of follow-up care up to the patient (PIFU) has been studied in RCTs [17–19]. The main principle in PIFU is to reduce inappropriate regular follow-up appointments. This by allowing patients to initiate hospital follow-up appointments on an ‘as required’ basis compared with the traditional ‘physician-initiated’ model [20]. PIFU with nurse involvement is found to be comparable to or better than traditional medical routine consultation, measured on both clinical and psychological factors [17–19]. Likewise, security was maintained with same level of decreased disease activity in PIFU guided by tight control of disease activity without affecting patient satisfaction, even among newly diagnosed patients, compared to traditional outpatient consultation every 3–4 months [21].

Internationally, there is a growing interest in how healthcare systems can be more flexible and individual-oriented, and patients with lifelong diseases are increasingly involved with their own treatment and care [22]. In addition, there is concurrent awareness that patients’ own ability to manage their lifelong disease may be an overlooked resource, and hinder patients from significant influence over their own treatment and care [23, 24].

From both a general political point of view and that of healthcare managers, there is growing awareness of the value of creating a health care system that ensures patient-centred care, where patients receive the necessary support for their individual and fluctuating needs related to a lifelong and treatment-demanding disease balancing costs as well as patient’s satisfaction and quality of life [20]. Involvement of nurses is supported by increasing evidence in a recent update of expert and evidence-based recommendations around the role of the nurse in the management of lifelong IA [25]. However, there is a need to investigate patients’ perspectives of various organizational changes and their experiences in relation to involvement in their own care. Therefore, the objective was to explore how patients with IA with low disease activity or remission (DAS-CRP < 2.9, a continuous measure of RA disease activity) experience PIFU, which is a way of involving patients in their own treatment and care.

## Method

### Design

This was a qualitative exploratory study based on focus group discussions among patients with IA, following a multidisciplinary decision of change from the traditional, fixed appointment system to a system of PIFU for the control of disease activity in a Danish outpatient clinic for rheumatology and spine diseases [26]. The study protocol was approved by the steering group for the PIFU project responsible for the implementation of PIFU. The Consolidated Criteria for Reporting Qualitative Research (COREQ) checklist was used to guide reporting [27]. Additional file 1 (Supplement S1) presents a completed COREQ checklist applied to this study.

### Clinical setting

PIFU was implemented in October 2016 in the outpatient clinic (inflammatory section) that includes a clinic led by rheumatologists seeing 13 patients each per day, and a nurse-led rheumatology clinic, which has up to 66 visits per day. The nurse-led clinic provides various services for the rheumatologist and patients at the outpatient clinic (Table 1). The reorganized follow-up care was based on a number of previous studies and subsequently adapted [13, 17, 18, 28]. The new set-up ensured a minimum recommended level of disease activity monitoring, while fulfilling the national monitoring plan as prescribed by the Danish nationwide clinical register for patients with rheumatoid arthritis (DANBIO) necessitating a minimum of two annual visits [4]. We implemented PIFU pragmatic leaving enrolment up to the rheumatologist and a snow-ball effect of the change in follow-up strategy.

**Table 1:** Usual arrangements versus Patient-Initiated Follow-Up (PIFU) in the rheumatology outpatient clinic

Care and services offered to patients with IA^a^							
Treatment characteristic 1) Conservative or unlicensed medicine (p.o.^b^) 2) Licensed medicine (p.o., s.c.^c^) 3) Drug infusion		Model of follow-up care					
		Usual			Patient-Initiated Follow-Up (PIFU)		
		1)	2)	3)	1)	2)	3)
**Disease monitoring**	Blood test	Every 8–12 weeks					
	PROM^d^ (DANBIO)^e^	Self-reported, on-site ahead of any consultation and/or drug infusion					
**Rheumatologist consultation**	Traditional medical consultation (*15 min*)	Fixed, planned every third to fourth month			Annual		
	Acute consultation	Significant waiting time (*5–8 days*)			Usual rheumatologist within five working days or any rheumatologist within 1–2 days		
**Nurse-led clinic**	Individual nurse consultation (*45 min, approximately six months offset from the medical consultation*)	Not offered (NO)			Annual nurse consultation; aiming to cover the patient’s knowledge of his/her actual situation, preferences and needs and includes screening for cardiovascular risk factors		
	Telephone support	Direct telephone to a nurse on weekdays from 1 to 3 pm and a secretary 8–12 am who can leave a message for a nurse or renew prescriptions			Hotline, manned on weekdays 8 am to 3 pm by an experienced RRN^f^		
	Drop-in function (e.g. *dispensing or administration of medicines and self-administration training*)	NO	Medication pickup with an interval of eight weeks (e.g.*, prefilled syringes*)	NO	NO	As usual	NO
	Infusion room (*Manned with 2–3 nurses on weekdays from 8 am - 3 pm*)	NO	NO	Drug infusion (*30–60 min*) administered by a nurse every 6–8 weeks	NO	NO	As usual
	Delegated tasks (*e.g, I.M.*^g^*injections, drug information, planning of treatment and laboratory test, infection detection*)	As usual					

*Abbreviations*: ^*a*^*IA* rheumatoid arthritis, psoriatic arthritis, axial spondyloarthritis, ^*b*^*p.o* per os, ^*c*^*s.c.* subcutaneous, ^*d*^*PROM* Patient-reported outcome measures, ^*e*^*DANBIO* The Danish nationwide clinical register for patients with reheumatoid arthritis, ^*f*^*RRN* Registered Rheumatology Nurse (educated between 1983 and 2005 with an average of 17 years of experience within rheumatology), ^*g*^*I.M.* intramuscular, *NO* Not Offered

### Participants

Inclusion criteria for PIFU were patients with low disease activity or remission (DAS-CRP < 2.9, from now low disease activity), ≥ 18 years of age and Danish-speaking without cognitive deficits (to ensure ability to take co-responsibility for own treatment to a sufficient extent). For the purpose of this study we added the following criteria: ≥ 6 months experience of PIFU who had received the additional annual nurse consultation and being able to participate in a focus group session in the hospital setting.

### Recruitment

Patients were recruited in two ways. Firstly, they were presented to the study as part of a patient satisfaction survey targeting patients enrolled in PIFU. In this survey, administered anonymously on a tablet prior to the annual nurse consultation, patients were also asked to upload their name and telephone number on a separate list if they were interested in participating in a focus group. Secondly, written information in the outpatient infusion room, encouraged patients to express interest by leaving their telephone number. A purposive selection was made among those patients who had agreed to participate after detailed information by telephone, performed by BB. The purposive selection aimed to achieve maximum variation among participants in relation to age, sex, type of diagnosis, treatment and disease duration [29]. We planned to invite 10–12 participants for each focus group and recruitment of participants was ongoing from June 2017 until March 2018 (Table 2). The written information was sent, either by e-mail or by regular post. Participants signed a consent form when they showed up to participate in one of the four focus groups in a staff conference room in the hospital.

**Table 2:** Recruitment and characteristics of the participants in the four focus groups

	Total	FG1	FG2	FG3	FG4
Agreed date of participation, n	37^a^	8	10	8	12
Notified cancellation^b^	10	1	1	3	5
Did not show up on the day^c^	3	1	0	1	1
Participants’, n	25	6	9	4	6
*Diagnosis, n:*					
RA (positive/negative)	21 (11/9)	4 (0/4)	8 (3/4)	3 (3/0)	6 (5/1)
PsA	3	2	0	1	0
axSpA	1	0	1	0	0
*Sex*					
Female, n (%)	20 (80)	6 (100)	4 (44)	4 (100)	6 (100)
*Age, years (mean [range])*	61.8 [28–79]	58.8 [47–62]	60.0 [28–76]	64.3 [51–74]	66.0 [51–79]
≥ *63 (67*^d^) *year , n*	14 (10)	1 (1)	6 (4)	3 (2)	4 (3)
*Years since diagnosis (mean [range])*	14.3 [4–59]	8.0 [4–16]	17.9 [7–59]	13.5 [9–22]	15.8 [4–28]
*Weekly work hours, n*					
Retired or working ≤16 h/week	17	5	5	2	5
Working ≥37 h/week	8	1	4	2	1
*Civil status, n*					
Married/cohabitant	18/2	4/0	6/1	4/0	4/1
Living alone	4	1	2	0	1
Did not answer	1	1	0	0	0
*Routine attendance in the nurse-led clinic*					
Licenced medication handed out every 8 weeks	13	6	6	1	0
Receive infusion every 6–8 weeks	6	0	3	3	0
Annual nurse consultation only	6	0	0	0	6

*FG* Focus Group, *n/N* number/Total, *%* percentage, *RA* rheumatoid arthritis, *PsA* psoriatic arthritis, *axSpa* axial spondyloarthritis

^a^One participant accepted dates for both FG1 and FG2 as she forgot and did not show up for the first focus group

^b^Reasons were Illness of own/kids/grandkids (n = 3/2/2), other priorities arose for the day (n = 2) and snow (n = 1)

^c^Unknown reason

^d^Average retirement age in Denmark in 2004 (2018)

### Procedure and setting for focus group discussions

A topic guide (Table 3) structured around follow-up care (Table 1) was developed by members of the steering group, including a patient research partner (PRP) diagnosed with RA who validated the topics and prompts. This topic guide was applied unchanged to all four focus groups. The aim of the focus groups was to facilitate a dynamic discussion on views on PIFU. Therefore, we sought to use the individual participants’ contributions to encourage all participants to share their own views and engage in the dialogue [26].

**Table 3:** Topic guide

Opening question: “What have your thoughts been about the new arrangements in your follow-up care?”	
1: PIFU^a^ – Patient Role	
How do you experience that your role as a patient has changed? Pro and cons?	
2: PIFU^a^ – Patient Value	
What are your expectations of needs that have to be met in follow-up care? What contributes to the feeling of being in control with your arthritis and treatment? E.g., what contributes to your feeling of being at the centre of decisions about treatment and needs?	
3: Annual Nurse Consultation	
What is your experience of the consultation with a Registered Rheumatology Nurse (RRN)? E.g., what needs are met? How do you experience the setting? E.g., need for same nurse, the providing of PROM and PROM use and value?	

^a^Patient-Initiated Follow-Up

To ensure confidentiality, none other than the moderator (BB) and co-moderator (BAE) were present with the participants who were asked to respect the confidentiality of all group members and all participants agreed. Prior to the focus group, the moderator and co-moderator introduced themselves, their professional background, including experience from different fields of nursing (15–29 years), within rheumatology (1–6 years) and research (2–14 years), their specific interest in the research topic, and background for the study. Initially, the moderator started with a broad, open-ended question such as “What have your thoughts been about the new arrangements in your follow-up care?” Probes like “Please give an example” or “Please explain in more detail” were used to nuance the answers and deepen the discussions. The co-moderator (BAE) assisted in digitally audio recording the focus group discussions, observed group interactions, and contributed with clarifying questions.

### Analysis

**Fig. 1:**
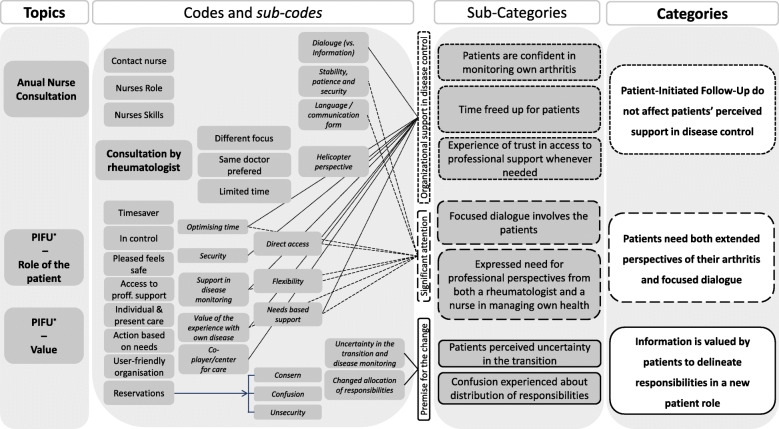
Coding tree illustrating the structure in the analysis with topic, major codes and categories. *PIFU: Patient-Initiated Follow-Up

After each focus group, a short debriefing between the moderator and observer summarized field notes taken during the discussion and initial impressions were added. The audio recordings were uploaded to a secure server and transcribed verbatim by a secretary compliant with the transcription of audio recordings. All names and personally identifiable information in the transcripts were deleted to comply with Danish law. BAE and BB each listened independently to the recordings and reviewed each transcribed text several times to ensure the quality of the transcription and get familiarized with the data. All four transcripts were then uploaded to NVivo (version 11, QSR International) to facilitate a structured analytical process based on qualitative content analysis, which was applied across all four transcripts [30, 31]. Initially, all meaning units were coded deductively by BB, based on the contents in the topic guide (Table 3). This part of the analysis focused on the manifest content (the visual and obvious content, ‘What the text says’) [30]. Next, the two researchers (BB and BAE), experienced within various qualitative methods, met to discuss the initial coding. During the subsequent analysis, BB and BAE had regular meetings to ensure consensus on the gradual condensation of subcategories to more overall categories. In this part of the analysis, the new coding of the four preliminary units of analysis was coded again with a focus on the latent content (interpretations of what was said, ‘what the text is talking about’) [30]. In this step we also invited the PRP to comment on the research findings. Through discussions and comparisons of similarities and differences, a consensus on the final categories and subcategories was reached. The analytic process is illustrated in Fig. 1. During the analysis, the authors agreed that the investigated topics was saturated in the four focus groups. To enhance validity the PRP was actively involved during the entire scientific process when; 1) the idea for the study was generated, 2) identifying and prioritizing content for the topic guide, 3) feedback was given on the protocol and written patient information, 4) analyzing the transcribed interviews and in the interpretation of the findings 5) commenting on the draft manuscript and final approval. This collaboration was based on the principles of respect and equality between PRP and researchers [32].

## Results

In total, 50 out of 193 invited participants indicated an interest by allowing a phone call for further information. Three participants were not informed further because we were not able to make contact to them (*n* = 2) or because of the purposive selection (treatment characteristic already well represented/full booked group, FG4) (*n* = 1). After receiving information by phone, three patients refused participation. Of 44 participants willing to participate, 37 accepted dates for participation of whom 25 participated (Table 2). Twenty (80%) were female, mean age 61.8 [range 28–79]. The four focus group discussions lasted on average 85 [range 74 to 104] minutes. The analysis resulted in three categories and seven subcategories; (Table 4). Numbered quotes (PxFGx, Participant number and Focus Group number) support the result.

**Table 4:** Overview of results

Categories	Subcategories
Patient-Initiated Follow-Up do not affect patients’ perceived support in disease control	• Patients are confident in monitoring own arthritis • Time freed up for patients • Experience of trust in access to professional support whenever needed
Information is valued by patients to delineate responsibilities in a new patient role	• Patients perceived uncertainty in the transition • Confusion experienced about distribution of responsibilities
Patients need both extended perspectives of their arthritis and focused dialogue	• Focused dialogue involves the patient • Expressed need for professional perspectives from both a rheumatologist and a nurse in managing own health

### Findings

#### Patient-initiated follow-up do not affect patients’ perceived support in disease control

Over time, several patients experienced a reduced need of frequent visits to their rheumatologist because of their knowledge of the symptoms and treatment effects. The change in scheduled consultations by a rheumatologist from every 3–4 months to one consultation with a rheumatologist followed by a consultation with a nurse 6 months later, was described as a sensible change and optimization of available resources that would benefit patients. Optimization of time contributes to the experience of a user-friendly organization*. “...It becomes more user-driven, because, if I am fully satisfied by talking to the nurse… then the doctor can concentrate on what he needs to do ... In this way the hospital’s resources are better utilized, I think...”*.(*P5FG2)* Direct access to professional support when needed meant the participants were at the centre and the flexibility provided security in the new arrangements. The two annual consultations and the continuous monitoring of the arthritis gave an experience of continued disease control.

##### Patients are confident in monitoring own arthritis

The participants were familiar with their arthritis. Some had, on an unconscious level, developed strategies to manage their disease*.* The participants had experience with stability in their arthritis and felt this was necessary in order to know what to be expected from the arthritis and when to act accordingly to PIFU. *“…after a number of years, you know your illness, become more familiar with the symptoms, and you know when it passes a certain level in some way, you know? This requires action in one way or another, right?...”(P4FG3)* For some, knowledge of their rheumatic fluctuations reduced the need for regular medical visits. Others felt that the prescribed medical visits were mostly for the rheumatologist’s sake. The continuous regular check of blood samples and X-rays created a sense of security for the effect of treatment. Some went on the Internet (Sundhed.dk; an option available to all Danish citizens with Social Security Number) and checked their own test results and thus had control of their blood samples themselves. Therefore, they did not see any reason to consult the rheumatologist in order to know whether their blood tests were normal. Participants felt confident, because several of them had experienced that action was taken if a blood test result caused concern. This gave them the impression that the situation was still under control, even with the new arrangements.

##### Time freed up for patients

The previously scheduled consultations were unnecessarily time consuming. *“...I sometimes feel that I am wasting both my own and the doctor’s time ... So, in that way, I think it* [PIFU] *was beneficial ... in that way, it was both the doctor’s and my own time, that was saved...”.*(*P1FG1)* Participants had a positive expectation that the new arrangements allowed rheumatologists and nurses to take on more important tasks, like the more acute ones. *“...I don’t have to talk about a nice party or something like that. But on days where you are bad, then it’s very nice to have some time...”.(P1FG3)* In comparison to the rheumatologist consultation, the participants appreciated the experience of more time at the annual consultation with the nurse. Several expressed a confidential relationship with the nurse and that the continuous contact covered their needs.

##### Experience of trust in access to professional support whenever needed

The participants were confident that they would receive help, if needed and emphasized that the patient knows best when there is need for consultation. In particular, it was important that they could easily access the nurses by using the direct telephone number and have access to consultation whenever the need arose. The participants knew that their questions would be answered by the rheumatologist and they experienced the new arrangement as following the patient’s needs rather than the rheumatologist’s. It was not essential to have an acute appointment in the outpatient clinic with either of the rheumatologist. However, frustration was expressed about the fact that different rheumatologists saw them for scheduled outpatient visits. Participants felt more secure when followed by the same rheumatologist. *“You get the impression that doctors, just like hairdressers, don’t all do things in quite the same way ... you would like to not suddenly have it done in a completely different way, and then have to get back on track the next time...”.(P5FG2)* The two scheduled appointments in PIFU were compared to a safety net by several participants, but some participants did not really have the need for these two consultations. For example, one participant indicated being able to cope as long as ‘one’s case’ remained open, with the opportunity to make contact when necessary. Several stated that having a telephone number may be enough, when ‘everything is going smoothly’ and there is no pain.

#### Information is valued by patients to delineate responsibilities in a new patient role

The various information about transition to PIFU gave rise to doubts about the continuous monitoring, patients’ opportunity to take action and whether there really had been a choice in relation to entering into the new arrangements. The participants positively welcomed a more active role as a patient but pointed out that patients should be well-informed and willing to take on a more active role. *“...if I am going to take responsibility and be a part of it, then I have to be well-informed ... The nurse, she helped to inform me of some of the things that is difficult to me ... Do you want this or that medicine? Do you? Or, e.g., do you want to attend the PIFU? Yes, but then tell me something more about what that actually means? Do you want this medicine or biological? If I have to decide, well then, I need to know more, right. If I have to take responsibility, then I have to base it on something...”.(P5FG4)* At the same time, many expressed their concern about a potential social downside, given that PIFU may not suit all patients.

##### Patients perceived uncertainty in the transition

Information given and dialogue about the new arrangements at the outpatient clinic varied and were variously described: as sufficient – in the sense that the patient did not feel abandoned – or as inadequate or completely absent. It gave rise to thoughts and concerns, and for some it brought uncertainty. For example, there were considerations in relation to the plan for the scheduled appointments, the interval between blood tests and whether the blood test would be seen by a rheumatologist. Some form of continuous feedback and a yearly plan with the scheduled appointments were requested. Several had also been confused by how bad one’s state should be before phoning. *“...I’ve had that feeling of, can I phone now or what is it about? When can I do it and how bad should my condition be and things like that, so you are uncertain? I have been uncertain about it at least, what it entails...”.(P3FG3)* Some had also been uncertain about what they could expect from the consultation with the nurse, and those who had not yet needed emergency assistance expressed an enthusiastic anticipation about the possibility of getting an acute appointment as promised and also the consequences of any future cost savings.

##### Confusion experienced about distribution of responsibilities

Participants felt a greater responsibility in PIFU and expressed concerns about whether all patients would call for help when needed. They also felt obliged to become more alert as a patient; for example, if their prescribed tests were not followed up on or even ordered. *“... if you are used to it, then you question why you suddenly don’t have* to [have blood tests]*, and then you doubt whether you should or not?... But if you are new to it then you don’t know that it is something you should do, right...”.(PXFG4).*

The expectation that patients in PIFU have to manage their own treatment process to a greater degree was discussed. Participants did not believe that the responsibility for the treatment should be removed from the rheumatologist or nurse onto the patient. They considered the thought to be frightening and they had, e.g., the notion that the blood test results should still be assessed by the rheumatologist. Also, they expected the rheumatologist to be in continuous control over the dosage of medicine and when X-rays were needed, etc. *“... there are some professional norms and standards that must be followed in order to monitor whatever kind of disease it is. And to my understanding there is now room for patients to get involved in the process and to say ‘Oh, it’s a bit difficult right now’ and then it will be taken care of. That balance is important...”.(P8FG2)* They agreed that it is the patient’s own responsibility to get blood tests done and see the rheumatologist and nurse as agreed. *“... you have responsibility to proactively contact your doctor and say, “Now I am fine”, “Now I am bad“, so the doctor has enough information to give the right treatment...”.(P5FG2).*

#### Patients need both extended perspectives of their arthritis and focused dialogue

The interaction with professionals, care, interest and focus on the individual problem made the participants feel that they were the centre of attention. For example, when the rheumatologist showed interest in other, non-related arthritis problems and when the nurse, e.g., involved the patient in the care by showing the blood test results directly on the screen, then the participants felt like co-players. The consultation with the nurse was referred to as valuable and the ongoing dialogue was also emphasized. *“...A consultation with the nurse is valuable because it is more than a snapshot* ... *the screen is a snapshot and the rheumatologist is a snapshot, but the consultation with the nurse is beyond this...”.(P5FG4).*

##### Focused dialogue involves the patient

In the dialogue with the nurse participants felt acknowledged. As an example, it was mentioned that the nurses reflected upon the participants’ answers in DANBIO and the participants received guidance where the nurse, among other things, addressed side effects and evaluated alternative options for, e.g., drug administration. The dialogue made participants feel supported in their illness and facilitated insight and coherence in their own treatment trajectory. *“...The conversation about what it means in regard to work, what it means in regard to fatigue, what it means. That you are acknowledged in your challenges. Or you can also say to experience the caregiving. Not just being a patient. I actually have a life with this. I felt it was really nice and meant that I got something else out of it* [the consultation with the nurse]*. Something I can use to move on in my life when I walk out the door…”.(P5FG4)* In addition, the participants expressed trust in seeking detailed explanation in the dialogue with the nurse. *“...you can talk about big or small issues without having to act on it right away ... it’s easier to just have a conversation with the nurse where, in a more non-committal way, you can slowly figure out what you need to be aware of…”.(P4FG1).*

The participants also recognized that the nurse was aware of a varied need for motivation and support. *“...my husband was diagnosed with leukemia ... they* [the nurses] *addressed the issue straight away. And I think that it’s really, really nice because the conversation was not about my rheumatoid arthritis but everything else”.(P2FG3).*

##### Expressed need for professional perspectives from both a rheumatologist and a nurse in managing own health

The combination of the rheumatologist and nurse throughout the treatment course in PIFU was something positive because of the experience of interaction in the nurse consultation*. “...I think it’s important to have the interaction...”.(P5FG4).*

The nurse consultation provided an outlet for frustrations and concerns beyond the actual disease and the effect of the medical treatment. The nurse was described as accommodating, present and caring, which contributed to conversations about key issues, and the opportunity to discuss more specific plans of action. The participants felt that the nurse had an overview of the patient’s entire situation. At the same time, there was an expectation that nurses should be able to spot deviations in the patient’s condition/health and assess any need for follow-up. It was positively received when, e.g., the nurse spoke about The Five Lifestyle Factors (1) healthy diet, 2) never smoking, 3) moderate-to-vigorous physical activity (at least 30 min/day), 4) moderate alcohol consumption, and 5) healthy body-mass index) with the patient in a different way than when the rheumatologist asked about problems related to arthritis. *“...when you have the other appointment* [with the nurse], *then it’s about something else. With the doctor you get your blood test checked, x-ray checked, you’re checked for everything and good facts ... Maybe there is more focus on the soft values when sitting there with the nurse … then you are, kind of, heard in another way … that it is not just about your arthritis. One also like having the slightly broader talk and I think that the nurse might be a little better at having a broader view than the specialist...”(P2FG3)* There was a specific need to talk to the rheumatologist when experiencing a downward curve or when things became complex in relation to medicine adjustment. The scheduled appointments with the rheumatologist also ensured an overall overview of the medicine. “*…I believe that it is two different things … I actually benefit from visiting the doctor once a year, because of the longer perspective...”.(P8FG2).*

## Discussion

All participants in our study underwent the experience of being transferred from routine care to PIFU with mainly positive experiences. Over the years they had experienced decreased need for close medical control. Despite well treated arthritis, our participants expressed a need to feel secure, and welcomed increased involvement in own disease control. Similar favorable experiences have been described by others, in a shift towards self-management and greater empowerment of patients with RA [15]. Our participants highlighted both the flexibility of PIFU and the opportunity for a broadened dialogue, as it contributed to individual support and the perception of person-centred care.

Focus of interest across Europe to reorganize the overall treatment of patients with IA has primarily been based on the expected shortage of rheumatologists, an expectation of increased number of patients with IA in the future, and discussions about the role of nurses in rheumatology. However, our project was not established with the intention of reducing costs or because of a shortage of rheumatologists. It was inspired by a strong political wish to achieve sustainable solutions by involving patients with lifelong diseases in their own disease course. Participants gave spontaneously feedback that they were satisfied with the new way of organizing follow-up care. It shows that it was possible to maintain the confidence of the patients in the changed follow-up care. This has also been found in a systematic review as crucial in the implementation of a Direct Access review system [15]. In addition, our participants reflected that PIFU was timesaving for both them and for the system. They also reflected on the previously scheduled consultations with the rheumatologist as resource intensive as they felt a need for more time, e.g., in periods with non-response to treatment or for those who are newly diagnosed. Accordingly, it is important to emphasize that changes in the healthcare system that aim to improve the patient’s situation can, at the same time, contribute to the positive redistribution of existing resources in favor of both patients and HPRs. However, fewer well-treated patients in the rheumatologist daily workflow meant reduced flexibility in the daily program. This consequence should be considered if planning similar organizational changes. Also, PIFU may be for the mentally strong and/or educated patient while e.g. lezz social capital can necessitate different requirements to follow-up care. Therefore, to hinder health inequality, another important aspect to be considered is deliberately selected inclusion criteria together with available support in follow-up care for those patients not fulfilling these criteria. This emphasizes a need to develop different forms of follow-up care to hinder a potential social downside mentioned by several participants in this study.

In this setting ongoing dialogue between patient and HPRs was ensured by yearly consultations with both a rheumatologist and a nurse. Aside from a reduction in scheduled consultations, the main difference for the participants in this study was the annual consultation and access to a nurse whenever needed, as recommended by EULAR [25]. This contrasted with the previous single professional program based on medical consultations. The participants welcomed the continued access to a nurse. Their positive evaluation was based on the different approach nurses took to working with the arthritis as some of them had experienced a narrow medical focus in traditional consultations also described by others [33, 34]. The nurse managed to reflect on the patients’ answers to the standardized PROM questions with the aim of addressing what matters to the patient now. Participants in our study emphasized that, when the nurse was actively involved in an in-depth dialogue, the nurse managed to target action to their current needs, as is recommended [25]. Participants also emphasized, e.g., the importance of a more reflexive dialogue about symptoms (not just medication) where the expertise of others also was considered. Like internal dialogue, others have described external dialogue as part of the health-promoting self-care of people living with RA [35]. External dialogue takes advantage of the individual’s personal and social resources [35]. Dialogue contributes to patients’ experience of participation, as they ‘experience involvement’ through the exchange of information and respect for their own knowledge and skills [36]. In previous studies, patients have emphasized the importance of dialogue with a nurse [16, 36]. Our participants also emphasized the dialogue with a nurse to be of importance despite the simultaneous course of the rheumatologist. Also, flexibility was assured in this set-up. Our participants expressed that it adds to the experience of a person-centred care when they can consult either the nurse or the rheumatologist whenever they experience the need. Gaining security, regularity, continuity and accessibility makes it easier for patients to solve current problems by themselves and to make decisions about what is needed and what can be done [16, 34]. Furthermore, flexibility also supports self-care by taking advantage of the individual’s personal and social resources [35].

In nurse-led clinics, more person-centred care has been argued as contributing to higher satisfaction with care based on an interrelationship and availability of the consistent care provider – compared to rheumatologist consultations only [24, 37–40]. Likewise, the nurses in our study were described as accessible, which the participants perceived as accessibility and “time for me”. The easy accessibility in the ‘easy to talk to way’ and/or attention has also been described by others [16, 34, 41]. Our participants had an experience that the nurse gave time and space and supported them in dealing with their actual situation. Duration of consultation is a factor known to influence patient satisfaction [42, 43]. The participants in our study were not aware of the exact difference in the time slots (45 vs. 15 min) for the scheduled consultations. However, they highlighted the experience of more time available in the consultation with the nurse compared to the rheumatologist. This is in line with findings from both a Swedish and a Dutch qualitative study [16, 41]. In consultations with more time available, emotional factors seem to be addressed with a more thorough assessment of the patient’s disease status and need for self-management support, i.e. psychosocial concerns, pain, fatigue, work/life balance provide patients with the experience of receiving more person-centred care [44, 45].

Despite an obvious need to feel safe about the treatment, the participants expressed openness to organizing practice in a different way, if they were not uncertain about what was expected of them. Insecurity was experienced as being related to concerns about the lack of resources, lacking information and absence of dialogue. A recent cross-sectional study found that dialogue with both a rheumatologist and a rheumatology nurse is emphasized as of utmost importance, especially dialogue about arthritis, treatment, lifestyle, physical activity and symptom management [46]. It is known that both disease-related factors and individual factors influence these dynamic needs and that support from different sources is needed [45]. However, dialogue could also take place in other settings, such as organized talks with researchers, other patients and lifestyle experts [46]. This underlines the need to continuously develop new ways to meet patients’ fluctuating needs for information, dialogue and professional support in handling life with arthritis. This might as well include contributions of allied HPRs, e.g. occupational therapists, physiotherapist and psychologists.

### Strengths and limitations of the study

The exploratory approach of qualitative focus group discussions was useful as little was known about the topic [26]. The group interactions during the discussions helped the participants to explore their own points of view and encouraged a range of responses that provided valuable insights into their perspectives. We encountered many patients who were not interested in participating (*n* = 177), and they may have different experiences. Therefore, our participants may not be representative of all patients with experience of PIFU. It is well-known that men in general are underrepresented in research. In total, 20% of the sample were men, which is lower than expected when including also patients with PsA and axSpA. Although we used a purposive criterion-based sampling strategy, the three diagnoses were not evenly spread across the focus groups. However, we consider our results to have high external validity, since the four focus groups included patients with RA, PsA and axSpA and there were variations in age and disease duration. Triangulation within members of the steering group including a PRP, rheumatologists, and rheumatology nurses, enhanced the trustworthiness of the study. The multidisciplinary decision of change in professional support for patients in this setting was restricted to existing resources in the outpatient clinic and thereby limited to adding nurse consultations to existing medical follow-ups by rheumatologists. Although this decision was supported by evidence, it is a weakness of this study that not all relevant HPRs are considered when changing in direction of a biopsychosocial approach in follow-up care. In addition, contextual factors may influence the transferability of findings. Also, differences between HPRs and their perspectives within categories were not explored, as this was not the aim of this study, still an important research area subsequent studies can explore.

## Conclusion

In the conclusion of this study, it is necessary to recall that those included in this study had established IA with low disease activity or remission and were considered suitable for PIFU by a rheumatologist. Interaction with both rheumatologists and nurses, with enough time for dialogue, broadens patients’ perspectives, makes opportunities for action visible, and contributes to patients’ ability to participate in the management of their own conditions. Patients allocated to PIFU welcome flexibility and involvement in disease control, which contribute to the perception of having events under their own control. However, patients need relevant information to be able to act adequately in the new patient role. In addition, patients expressed a strong wish not to consult only a rheumatologist or a rheumatology nurse, but to consult *both*, because both professions complement each other and address the different needs of patients throughout their life with arthritis.

## Supplementary information

**Additional file 1.** The COREQ (COnsolidated criteria for REporting Qualitative research) checklist applied this manuscript is provided as additional material.

## Data Availability

The datasets used and/or analyzed during the current study are available from the corresponding author on reasonable request and according to Danish low.

## Abbreviations

axSpA: Axial spondyloarthritis
COREQ: Consolidated criteria for reporting qualitative research, a 32-item checklist for interviews and focus groups
DANBIO: The Danish nationwide clinical register for patients with rheumatoid arthritis
DAS-CRP: A continuous measure of RA disease activity with measures < 2.9 categorized as low disease activity or remission (referred as ‘low disease activity’)
FG: Focus group (numbered 1–4)
HPRs: Health professionals in rheumatology
IA: Inflammatory arthritis
I.M.: Intramuscular
PIFU: Patient-initiated follow-up
p.o.: Per os
PROM: Patient-reported outcome measures
PRP: Patient research partner
PsA: Psoriatic arthritis
PxFGx (numbered quotes): ‘Px’ refers to the patient number (1–9) in the individual focus groups and ‘FGx’ refers to Focus Group number (1–4)
RA: Rheumatoid arthritis
RCT: Randomized controlled trial
RRN: Registered rheumatology nurse
s.c.: Subcutaneous
