# Supplementary material for: Patient-Initiated Follow-Up (PIFU) as reorganized support for increased patient involvement – focus group discussions among patients’ with inflammatory arthritis
Source: BMC Rheumatol. 2020 Jun 30;4:44. doi: 10.1186/s41927-020-00143-6 (PMC7325086; doi:10.1186/s41927-020-00143-6)
Supplement: Supplementary file 1 — Additional file 1. The COREQ (COnsolidated criteria for REporting Qualitative research) checklist applied this manuscript is provided as additional material. [file 41927_2020_143_MOESM1_ESM.docx]

**Additional File 1**

Supplement S1: **COREQ (COnsolidated criteria for REporting Qualitative research) checklist** [1] **applied the manuscript entitled “*Patient-Initiated Follow-Up (PIFU) as Reorganized Support for Increased Patient Involvement – Focus Group Discussions among Patients’ with Inflammatory Arthritis”***

| Topic | Item  No. | Guide Question/Description | Reported in section |
| --- | --- | --- | --- |
| Domain 1: Research team and reflexivity | | | |
| ***Personal characteristics*** |  |  |  |
| Interviewer/facilitator | **1** | Which author/s conducted the interview or focus group? | The first (BB) and last (BAE) author facilitated all four focus groups. This is reported in the Method section/‘Procedure and setting for focus group discussions’ |
| Credentials | **2** | What were the researcher’s credentials? E.g. PhD, MD | This is reported in the Declaration section/‘Author contributions’:  *...BB, nurse specialist (RN and MSc in Health Science) and BAE, senior researcher (RN, MSciN, PhD) – both females without treatment or care responsibilities of the participants - designed the study, collected the data, undertook coding, data analysis and drafting of the manuscript...* |
| Occupation | **3** | What was their occupation at the time of the study? |  |
| Gender | **4** | Was the researcher male or female? |  |
| Experience and training | **5** | What experience or training did the researcher have? | This is reported in the Method section/‘Procedure and setting for focus group discussions’ (...*including experience from different fields of nursing (15-29 years), within rheumatology (1-6 years) and research (2-14 years), their specific interest in the research topic, and background for the study.*...) and in the Method section/‘Analysis’ (...*experienced within various qualitative methods*…) |
| ***Relationship with participants*** | | | |
| Relationship established | **6** | Was a relationship established prior to study commencement? | No.  There was no relationship between the researchers and the participants prior to the study. This is reported in the Declaration section/‘Author contribution’: *...without treatment or care responsibilities of the participants …* |
| Participant knowledge ofthe interviewer | **7** | What did the participants know about the researcher? e.g. personalgoals, reasons for doing the research | Prior to the focus group, the moderator and co-moderator introduced themselves, their professional background, including experience within rheumatology and research, their specific interest in the research topic, and background for the study. This is reported in the Method section/ ‘Procedure and setting for focus group discussions’ |
| Interviewer characteristics | **8** | What characteristics were reported about the inter viewer/facilitator? e.g. Bias, assumptions, reasons and interests in the research topic | Different professional backgrounds of the first and last author (moderator and co-moderator respectively) and being without treatment or care responsibilities for any of the participants (both first and last author). This is reported in the Method section/‘Procedure and setting for focus group discussions’ and in the Declaration section/‘Author Contribution’. The different clinical nursing backgrounds may have prevented bias related to an insider perspective and contribute to a greater understanding. To limit bias, we asked only open-ended questions (table 3 ‘Topic guide’), so as to allow unexpected but relevant topics to emerge in the discussions. Also, most steps in the analysis were performed both individually and as a team. This is reported in the Method section/‘Analysis’. Several meetings were held ensuring different perspectives of the researchers were discussed and negotiated to enhance confirmability. Also, a research partner was involved to insure interpretation of data. |
| Domain 2: Study design | | | |
| ***Theoretical framework*** |  |  |  |
| Methodological orientation and Theory | **9** | What methodological orientation was stated to underpin the study? e.g. grounded theory, discourse analysis, ethnography, phenomenology, content analysis | In the abstract we state that the analysis was based on content analysis. In the method section/‘Design’ we state the design is a qualitative exploratory study while the content analysis is described in more details in the Method section/‘Analysis’. |
| ***Participant selection*** |  |  |  |
| Sampling | **10** | How were participants selected? e.g. purposive, convenience, consecutive, snowball | Participants were initially approached in two ways in the outpatient clinic (during answering a survey or while receiving treatment). This is described in the Method section/‘Recruitment’. This sample selection was done to recruit participants with the best possible experiences of the research topic while adhering to Danish law and GDPR rules that do not allow look-up in journals for eligible participants to ask for participation. |
| Method of approach | **11** | How were participants approached? e.g. face-to-face, telephone, mail,email | Recruitment procedure are thoroughly described in the Method section/‘Recruitment’. Participants were initially approached in two ways in the outpatient clinic (during answering a survey or while receiving treatment). Participants received oral and written information. |
| Sample size | **12** | How many participants were in the study? | 25 (20% female) patients participated in one of four focus group discussions. Details—both total and for each focus group—are displayed in table 2 (‘Recruitment and characteristics of the participants in the four focus groups’) and described in the result section |
| Non-participation | **13** | How many people refused to participate or dropped out? Reasons? | Reasons and numbers (n=13) for dropouts of planned participation is described by the explanation text in the bottom of table 2. Reasons for refusing to participate (not allowing phone call or rejecting when called) was not attempted to obtain. However, numbers are stated in the Result section. |
| ***Setting*** |  |  |  |
| Setting of data collection 1 | **14** | Where was the data collected? e.g. home, clinic, workplace | The focus group discussions were held in a staff conference room in the hospital. This is stated in the Method Section/‘Recruitment’. |
| Presence of nonparticipants | **15** | Was anyone else present besides the participants and researchers? | Non other were present. This is stated in the Method Section/‘Procedure and setting for focus group discussions’: *...To ensure confidentiality, none other than the moderator (BB) and co-moderator (BAE) were present with the participants who were asked to respect the confidentiality of all group members and all participants agreed...* |
| Description of sample | **16** | What are the important characteristics of the sample? e.g. demographicdata, date | Characteristics of the sample are displayed in Table 2. PIFU was implemented October 2016 - this is stated in the Method section/‘Clinical setting’. Recruitment for the focus group discussions were ongoing from June 2017 until March 2018. This is stated in the Method Section/‘Recruitment’. |
| ***Data collection*** |  |  |  |
| Interview guide | **17** | Were questions, prompts, guides provided by the authors? Was it pilot tested? | Yes.  A topic guide with open-ended prompts was developed by the Steering group responsible for the implementation of PIFU. All authors, including a patient research partner who validated the topics and prompts, were part of this Steering group. This is reported in the Method section/‘Procedure and setting for focus group discussions’. |
| Repeat interviews | **18** | Were repeat inter views carried out? If yes, how many? | Yes. The topic guide (displayed in table 3) was applied unchanged to all four focus groups. This is stated in the Method section/‘Procedure and setting for focus group discussions’. |
| Audio/visual recording | **19** | Did the research use audio or visual recording to collect the data? | Yes. Digitally audio recording was used. This is stated in the Method section/‘Procedure and setting for focus group discussions’. |
| Field notes | **20** | Were field notes made during and/or after the interview or focus group? | Yes.  This is stated in the Method section/‘Analysis’: *...After each focus group, a short debriefing between the moderator and observer summarized field notes taken during the discussion and initial impressions were added...* |
| Duration | **21** | What was the duration of the inter views or focus group? | Yes. The four focus group discussions lasted on average 85 (range 74 to 104) minutes. This is reported in the Result section. |
| Data saturation | **22** | Was data saturation discussed? | Yes. During the analysis, the authors agreed that the investigated topics was saturated in the four focus groups. This is stated in the Method section/‘Analysis’. |
| Transcripts returned | **23** | Were transcripts returned to participants for comment and/or correction? | No.  Transcripts were not returned to participants for correction. Allowing participants to comment on the transcriptions is controversial within qualitative research. In our research group, we decided in advance that the participants should not be offered this opportunity. For the sake of the GDPR rules, we were required (from the Danish Data Protection Agency) to delete all names and personally identifiable information in the transcripts. Therefore, it would not make sense to hand over the transcripts to the individual participants, as they would not be able to identify their statements. |
| Domain 3: analysis and findings | | | |
| ***Data analysis*** |  |  |  |
| Number of data coders | **24** | How many data coders coded the data? | Two authors (the first and last author) coded the data. Details are described in the Method section/‘Analysis’. |
| Description of the coding tree | **25** | Did authors provide a description of the coding tree? | Yes.  The coding and the analysis is described in the Method section/‘Analysis’ and displayed in Figure 1 ‘Coding tree’. |
| Derivation of themes | **26** | Were themes identified in advance or derived from the data? | The sub-codes, subcategories and categories were derived from data (transcripts) through a gradual condensation of the latent content. Initial codes (manifest content) correspond to contents in the topic guide (table 3). The above is described in the Method section/‘Analysis’. |
| Software | **27** | What software, if applicable, was used to manage the data? | We analyzed the qualitative data using NvIVO. This is stated in the Method section/‘Analysis’: *...All four transcripts were uploaded to NvIVO (version 11, QSR International) to facilitate a structured analytical process...* |
| Participant checking | **28** | Did participants provide feedback on the findings? | No.  Participant checking has not been performed. In contrast the results have been presented and validated by a research partner. This is stated in the Method section/‘Analysis’. The research team had decided in advance not to allow the participants to comment on the final result. |
| ***Reporting*** |  |  |  |
| Quotations presented | **29** | Were participant quotations presented to illustrate the themes/findings?Was each quotation identified? e.g. participant number | Yes.  Each quotation are presented in the results section and identified by participant number (1-9) and focus group number (1-4), e.g. PxFGx. We had to relinquish from indexing quotes with gender, age or diagnosis due to ethical considerations as recruitment was from a single hospital. |
| Data and findings consistent | **30** | Was there consistency between the data presented and the findings? | Yes.  In the result section of the manuscript categories and subcategories are described in a structured manner. Included are quotations from different participants. |
| Clarity of major themes | **31** | Were major themes clearly presented in the findings? | Yes.  As data might have had mutual meanings, the authors made efforts to make the subcategories/categories mutually exclusive. The subcategories and categories are presented as to within similarities and between differences (Figure 1. ‘Coding tree’ and in Table 4. ‘Overview of results’). We chose to operate with categories and subcategories in relation to content analysis, and therefore did not operate with themes. We identified three categories and all of them have same value. Transferability was addressed by providing a detailed description of the context and setting (Described in Method section/‘Design’, ‘clinical setting’, ‘participants’ and Table 1.’ Usual arrangements versus Patient-Initiated Follow-Up (PIFU) in the rheumatology outpatient clinic’). |
| Clarity of minor themes | **32** | Is there a description of diverse cases or discussion of minor themes? | Yes.  All categories are discussed. Through our analysis work, we were concerned that there should be a clear connection between the purpose of the project and the transcriptions, and we were continually focusing on our results is based on data as described in the Method section/‘Analysis’. Of course, the analysis work consisted of a refinement of the wording in the categories (Figure 1. Coding tree and Table 4. Results). Still, as pointed out above, we believe that the three identified categories are apparent in both descriptions of the data and the selected quotes.The participants generally agreed with their experiences with follow-up care, and we are not aware of any outliers in the participants' statements. |

Reference:

1. Tong, A., P. Sainsbury, and J. Craig, *Consolidated criteria for reporting qualitative research (COREQ): a 32-item checklist for interviews and focus groups.* International journal for quality in health care, 2007. **19**(6): p. 349-357
